# Supplementary material for: Comparative Analysis of the CDR Loops of Antigen Receptors
Source: Front Immunol. 2019 Oct 15;10:2454. doi: 10.3389/fimmu.2019.02454 (PMC6803477; doi:10.3389/fimmu.2019.02454)
Supplement: Supplementary file 1 [file Data_Sheet_1.PDF]

Supplementary Material

1 SUPPLEMENTARY TABLES AND FIGURES

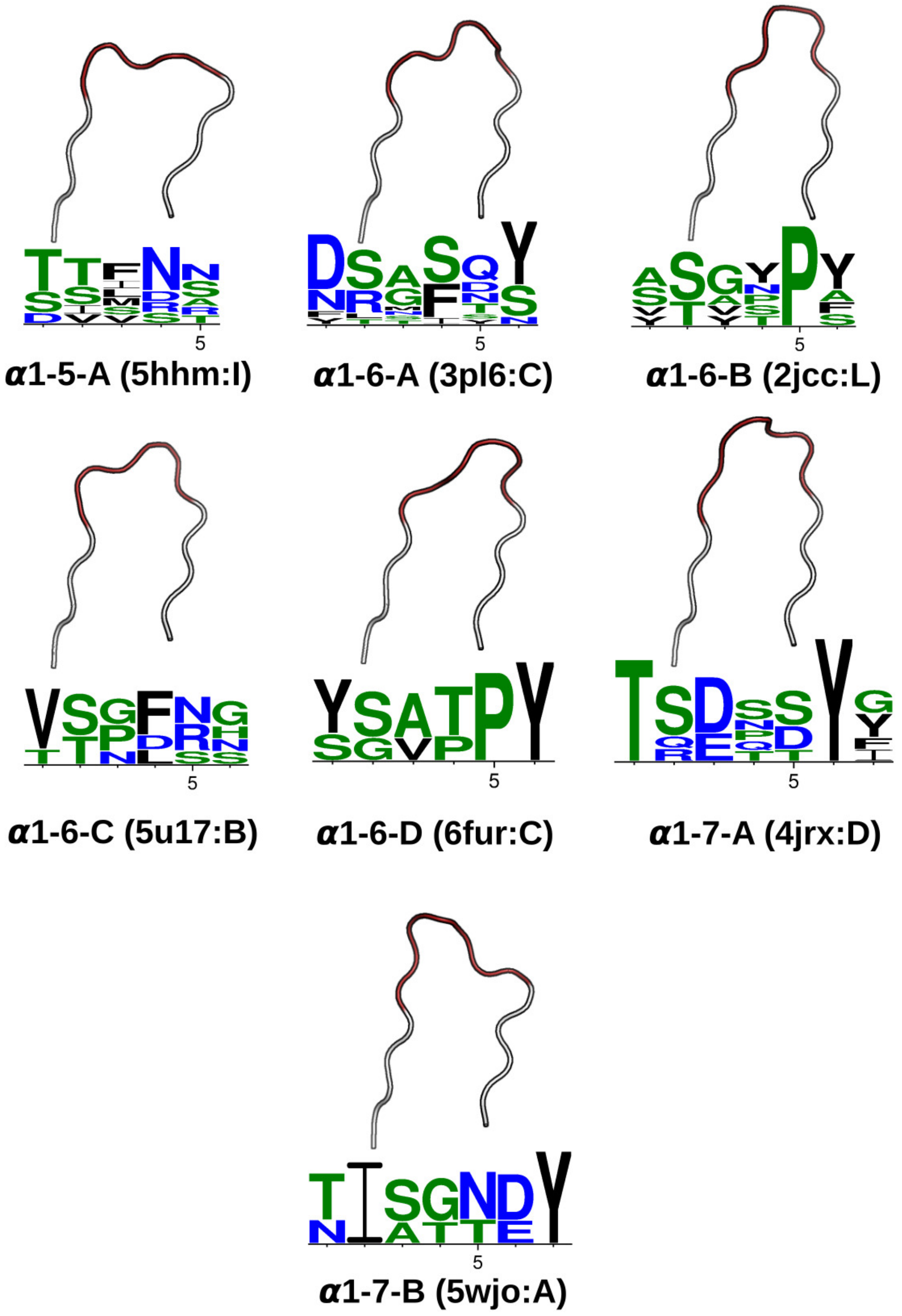

Figure S1: CDRα1 loop clusters. At a 1.0Å clustering threshold, our DBSCAN method identified seven canonical classes (see Results). Each class has at least five structures and two unique sequences. Anchors are coloured white, while the CDRα1 region (IMGT 27–38) is coloured. The Protein Data Bank (PDB; Berman et al., 2000) four-letter code and the chain identifier of the centroid structure is shown in the bracket next to the cluster name. The sequence pattern below each centroid structure is generated by WebLogo (Crooks et al., 2004), using the unique sequences of the cluster: α1-5-A has 7, α1-6-A has 13, α1-6-B has 6, α1-6-C has 5, α1-6-D has 3, α1-7-A has 6 and α1-7-B has 3.

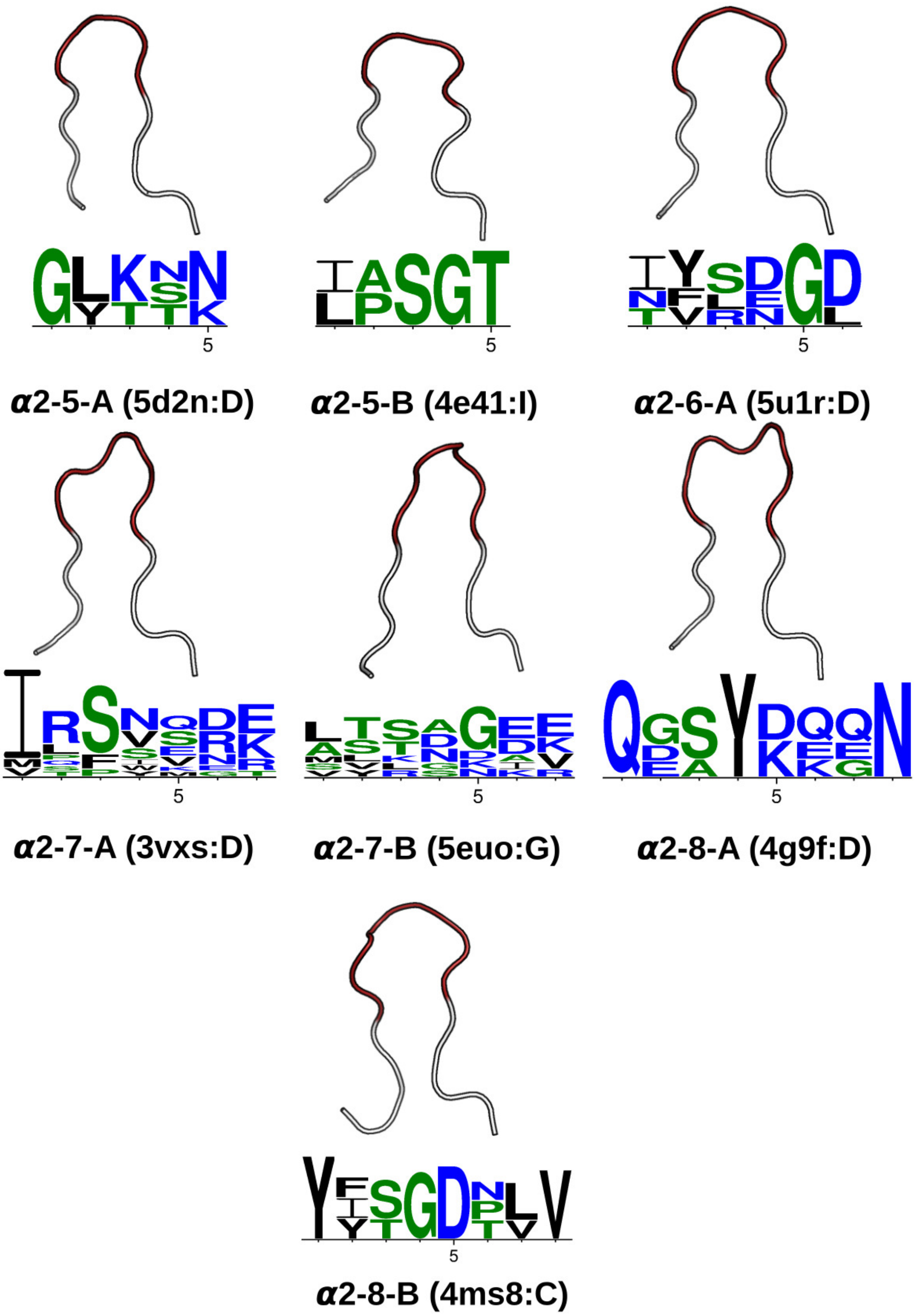

Figure S2: CDRα2 loop clusters. At a 1.0Å clustering threshold, our DBSCAN method identified seven canonical classes (see Results). Each class has at least five structures and two unique sequences. Anchors are coloured white, while the CDRα2 region (IMGT 56–65) is coloured. The PDB four-letter code and the chain identifier of the centroid structure is shown in the bracket next to the cluster name. The sequence pattern below each centroid structure is generated by WebLogo (Crooks et al., 2004), using the unique sequences of the cluster: α2-5-A has 3, α2-5-B has 2, α2-6-A has 4, α2-7-A has 12, α2-7-B has 8, α2-8-A has 4 and α2-8-B has 3.

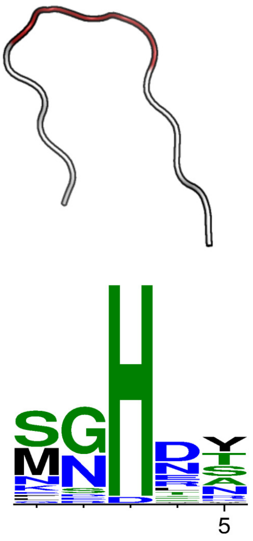

β1-5-A (1bd2:E)

Figure S3: CDRβ1 loop clusters. At a 1.0Å clustering threshold, our DBSCAN method identified one canonical class (see Results). Each class has at least five structures and two unique sequences. Anchors are coloured white, while the CDRβ1 region (IMGT 27–38) is coloured. The PDB four-letter code and the chain identifier of the centroid structure is shown in the bracket next to the cluster name. The sequence pattern below each centroid structure is generated by WebLogo (Crooks et al., 2004), using the unique sequences of the cluster: β1-5-A has 30.

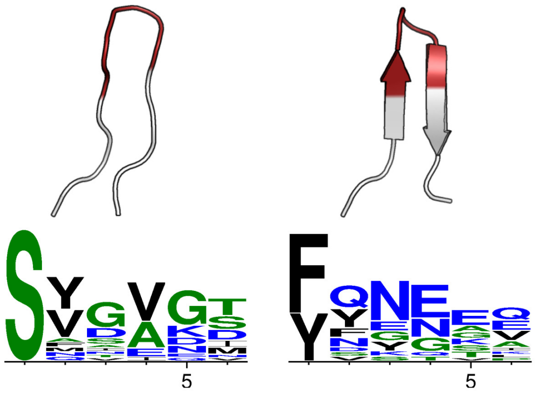

β2-6-A (3qh3:B)β2-6-B (3vxm:E)

Figure S4: CDRβ2 loop clusters. At a 1.0Å clustering threshold, our DBSCAN method identified two canonical classes (see Results). Each class has at least five structures and two unique sequences. Anchors are coloured white, while the CDRβ2 region (IMGT 56–65) is coloured. The PDB four-letter code and the chain identifier of the centroid structure is shown in the bracket next to the cluster name. The sequence pattern below each centroid structure is generated by WebLogo (Crooks et al., 2004), using the unique sequences of the cluster: β2-6-A has 18 and β2-6-B has 16.

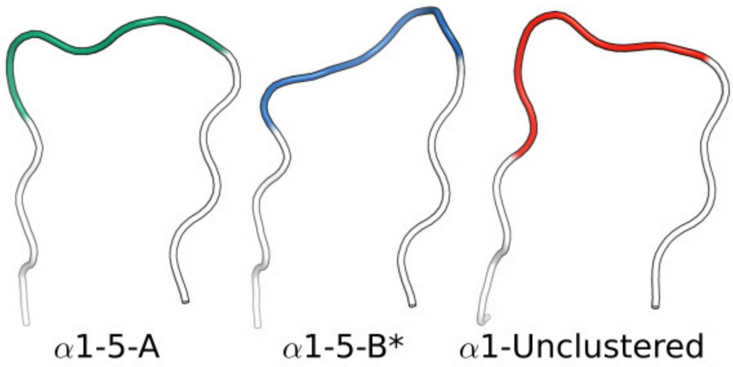

Figure S5: CDRα1 loops with the sequence DSVNN can form the α1-5-A canonical class (PDB: 5fka:A; unbound) or the α1-5-B* pseudo-class (PDB: 2ian:D; bound to MHC with a long peptide). Alternatively, they can also be unclustered (PDB: 4mnq:D; bound to MHC with a short peptide).

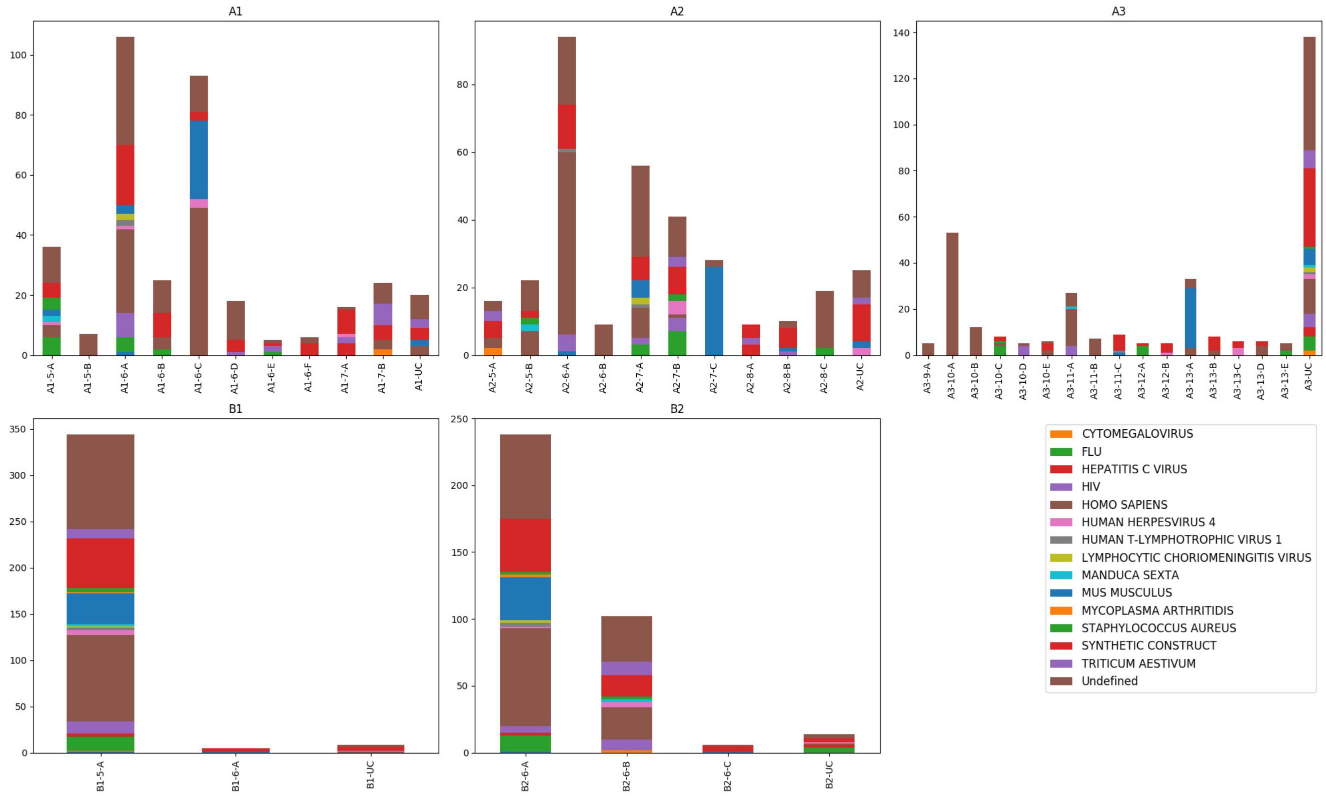

Figure S6: Antigenic peptide organism from the metadata of the TCR structures, sorted by CDR types and canonical forms.

Canonical class	Al-Lazikani	Klausen	Number of unique sequences (Number of structures)
α1-5-A		α1-1	7 (36)
α1-5-B*			1 (7)
α1-6-A	α1-1	α1-2	13 (106)
α1-6-B	α1-2	α1-3	6 (25)
α1-6-C		α1-4	5 (93)
α1-6-D			3 (18)
α1-6-E*			1 (5)
α1-6-F*		α1-5	1 (6)
α1-7-A		α1-7	6 (16)
α1-7-B		α1-6	3 (24)
(unclustered)	α1-3		
α1-unclustered			10 (20)
α2-5-A		α2-2	3 (16)
α2-5-B		α2-1	2 (22)
α2-6-A	α2-1	α2-3	4 (94)
α2-6-B*			1 (9)
α2-7-A	α2-2	α2-4	12 (56)
α2-7-B	α2-4	α2-5	8 (41)
α2-7-C*		α2-7	1 (28)
α2-8-A		α2-8	4 (9)
α2-8-B	α2-3	α2-6	3 (10)
α2-8-C*			1 (19)
α2-unclustered			11 (25)
α3-9-A*			1 (5)
α3-10-A		α3-3	11 (53)
α3-10-B*			1 (12)
α3-10-C*			1 (8)
α3-10-D*			1 (5)
α3-10-E*			1 (6)
α3-11-A		α3-5	6 (27)
α3-11-B*			1 (7)
α3-11-C*		α3-6	1 (9)
α3-12-A		α3-7	2 (5)
α3-12-B*			1 (5)
α3-13-A		α3-8	3 (33)
α3-13-B		α3-10	2 (8)
α3-13-C*		α3-9	1 (6)
α3-13-D*			1 (6)
α3-13-E*			1 (5)
(filtered)		α3-1	
(unclustered)		α3-2/α3-4/α3-11/α3-12	
α3-unclustered			72 (138)

Table S1. Summary of CDRα canonical classes. The CDRα canonical classes from our study are compared to the canonical classes of Al-Lazikani et al. (2000) and Klausen et al. (2015) (see Materials and Methods). Briefly, if there is at least one structure that is present in both our canonical class and those from the literature, we consider these classes to be analogous. Pseudo-classes are indicated with *.

Canonical class	Al-Lazikani	Klausen	Number of unique sequences (Number of structures)
β1-5-A	β1-1/β1-2	β1-1/β1-2	30 (344)
β1-6-A*	β1-3		1 (5)
(unclustered)		β1-3/β1-4	
β1-unclustered			3 (8)
β2-6-A	β2-1	β2-2	18 (238)
β2-6-B		β2-3/β2-4/β2-5	16 (102)
β2-6-C*	β2-3	β2-6	1 (6)
(unclustered)	β2-2	β2-1/β2-7	
β2-unclustered			6 (14)

Table S2. Summary of CDRβ classes. Clusters are labelled and mapped to previous canonical classes (see Table S1 and Materials and Methods). If there is at least one structure that is present in both our canonical class and those from the literature, we consider these classes to be analogous. Pseudo-classes are indicated with *.

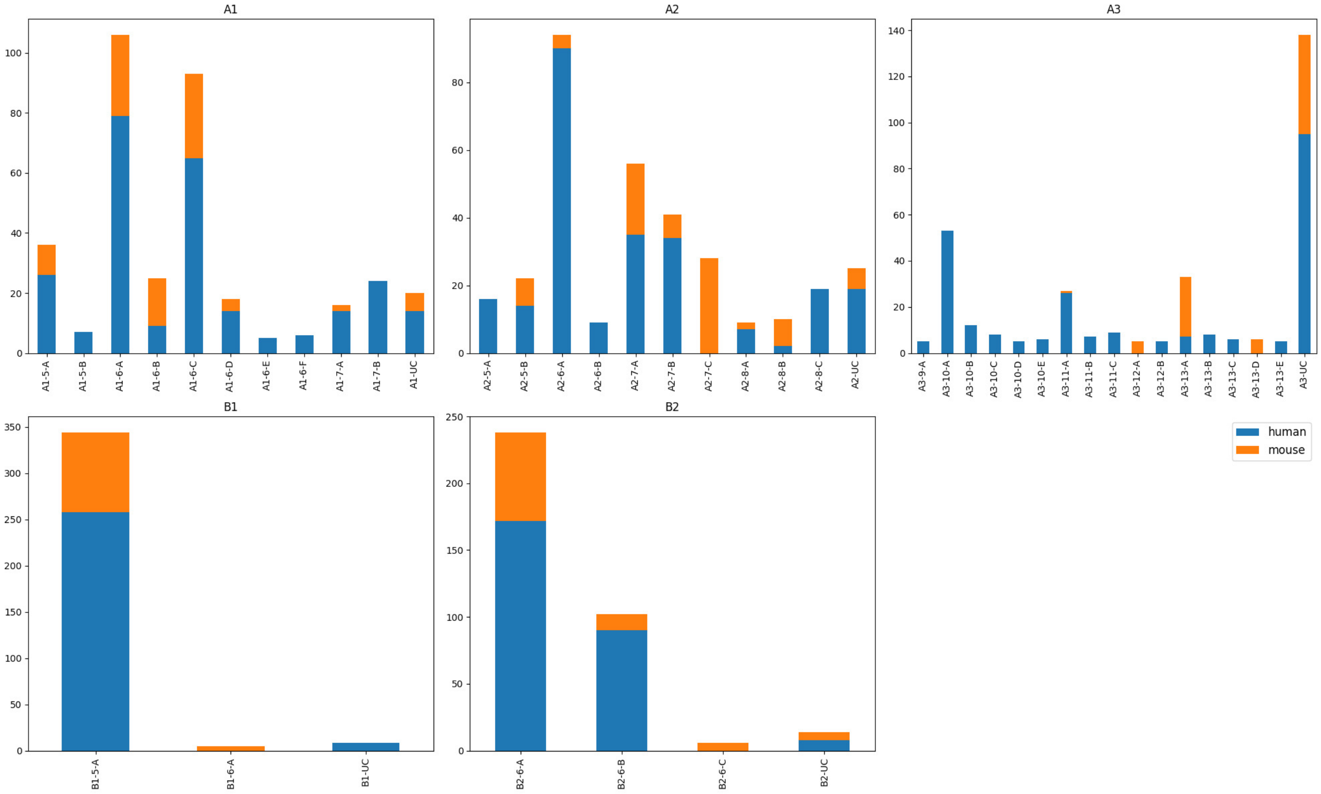

Figure S7: Species information from the sequence alignment of the TCR structures, sorted by CDR types and canonical forms.

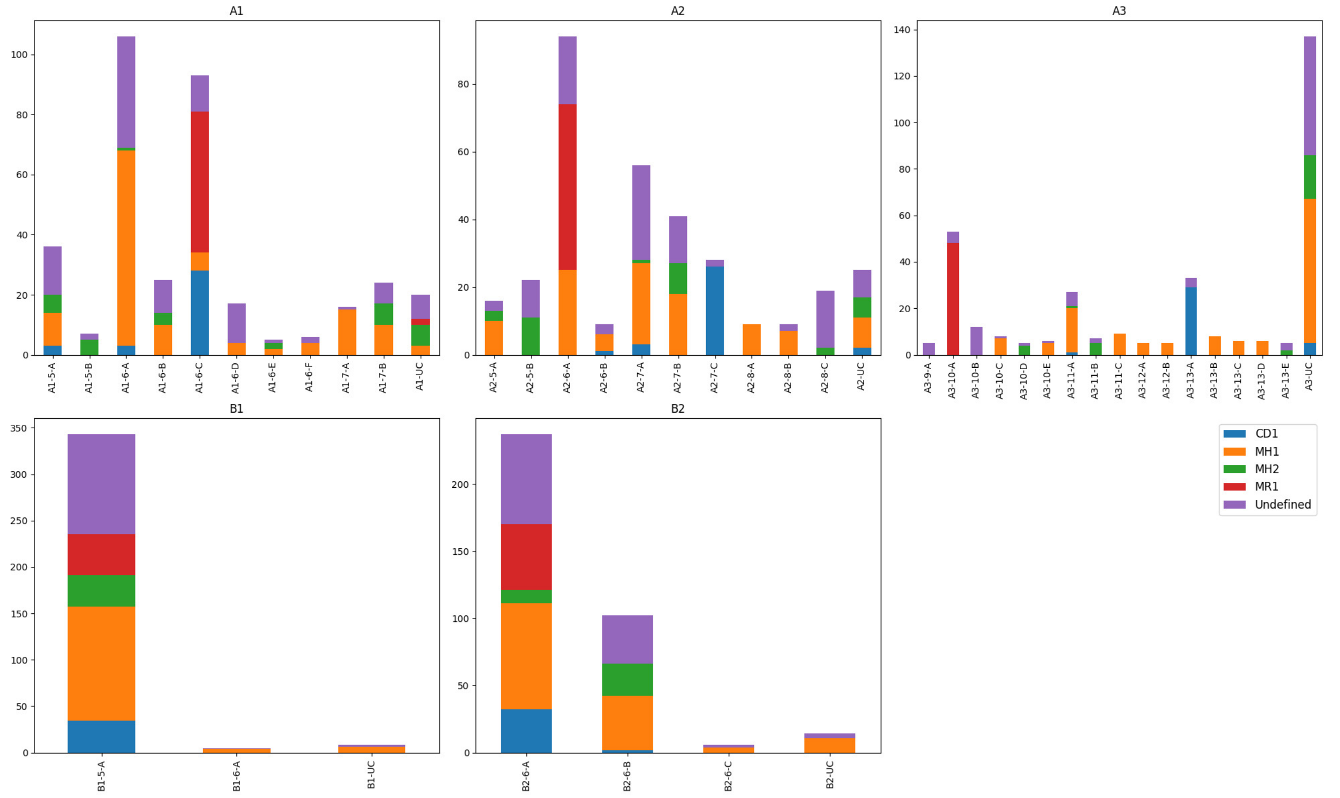

Figure S8: Types of major histocompatibility complex (MHC) from the alignment of the MHC sequences in the corresponding TCR structures, sorted by CDR type and canonical forms. CD1: Cluster of differentiation 1; MH1: MHC Class I; MH2: MHC Class II and MR1: Major histocompatibility complex class I-related protein.

Table S3. Number of sequences with multiple conformations.

	TCR and antibody (antibody subset)					
	L1	L2	L3	H1	H2	Total
# Unique sequences	580	198	855	793	885	3311
# Unique sequences with multiple structures	362	151	527	503	546	2089
# Unique sequences with structures in multiple classes	25	12	17	42	70	166
	TCR and antibody (TCR subset)					
	$\alpha1$	$\alpha2$	$\alpha3$	$\beta1$	$\beta2$	Total
# Unique sequences	45	44	99	33	39	260
# Unique sequences with multiple structures	39	35	72	30	36	212
# Unique sequences with structures in multiple classes	11	6	9	3	12	41

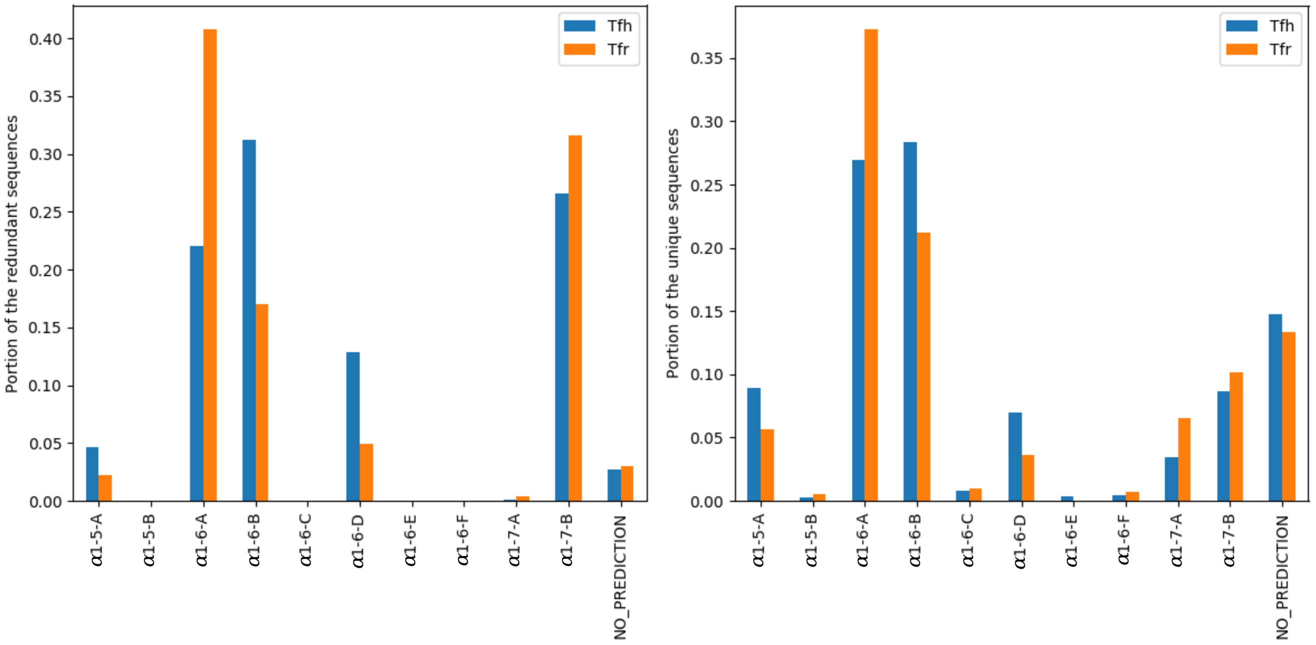

Figure S9: CDRα1 canonical form prediction from the TCR sequences in Tfh and Tfr cells from Maceiras et al. (2017).

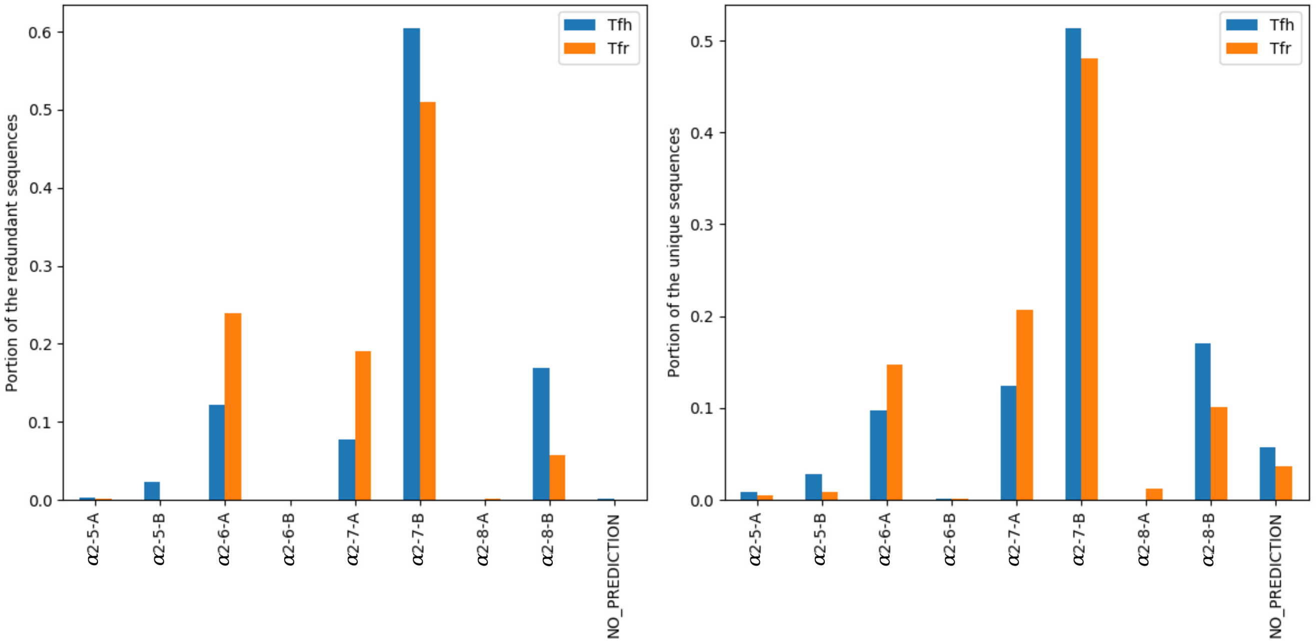

Figure S10: CDRα2 canonical form prediction from the TCR sequences in Tfh and Tfr cells from Maceiras et al. (2017).

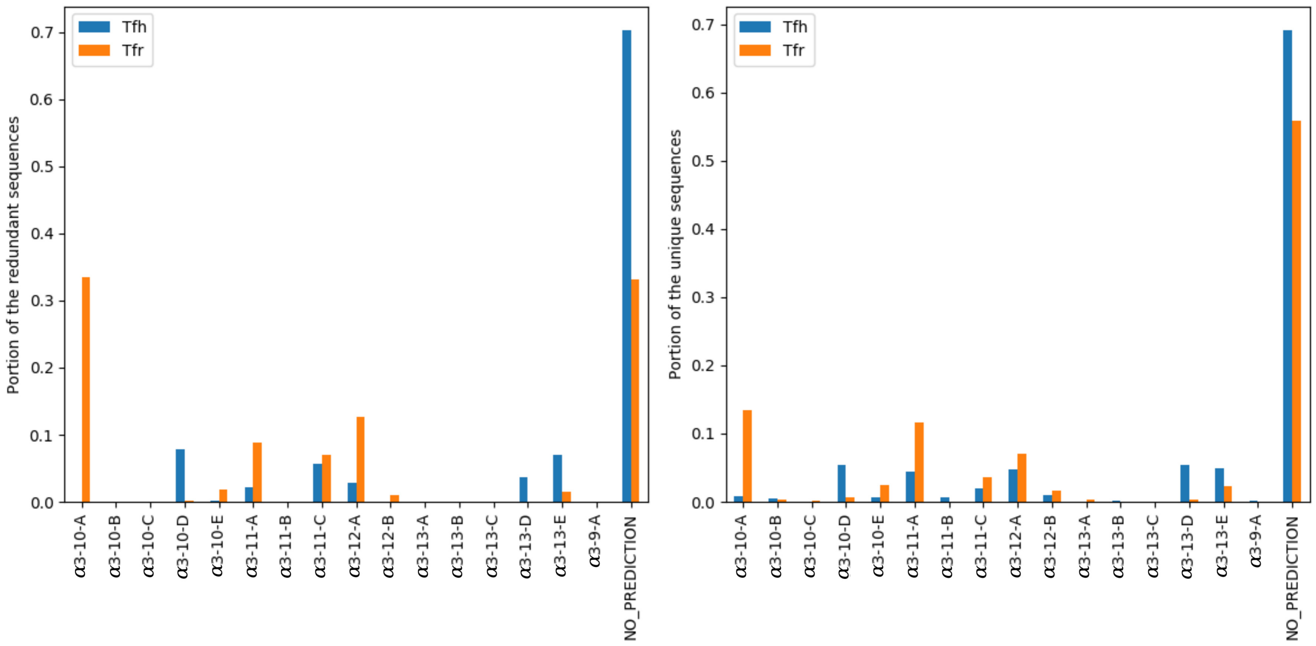

Figure S11: CDRα3 canonical form prediction from the TCR sequences in Tfh and Tfr cells from Maceiras et al. (2017).

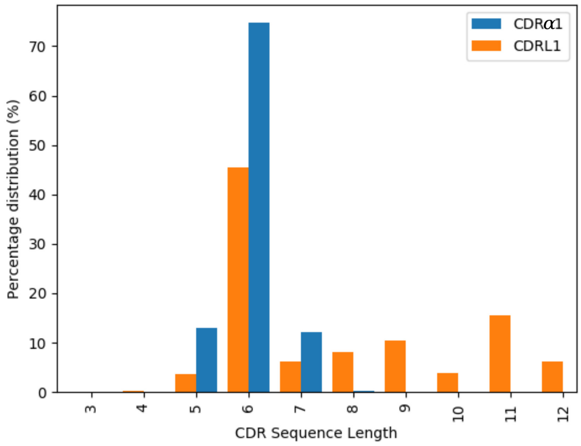

Figure S12: Length distributions of CDRα1 (blue) and CDRL1 (orange) loops. The length distributions overlap between CDRα1 and CDRL1.

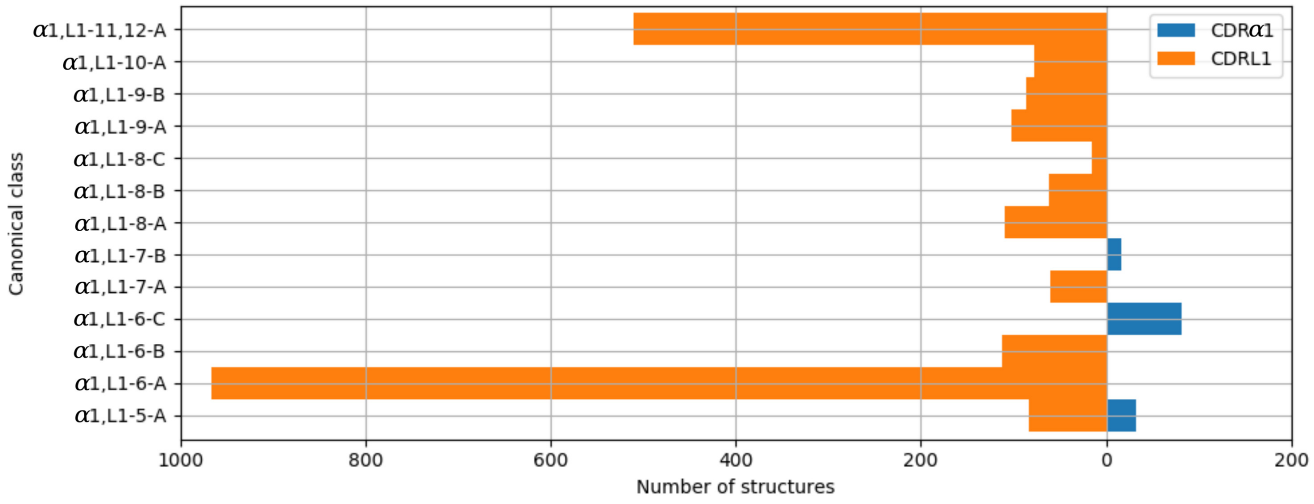

Figure S13: Clusters of CDRα1 (blue) and CDRL1 (orange) loops. Orange bars indicate the number of CDRL1 structures, and blue bars the number of CDRα1 structures. All classes, apart from α1,L1-5-A, have structures from only one CDR type, *i.e*. CDRα1 or CDRL1.

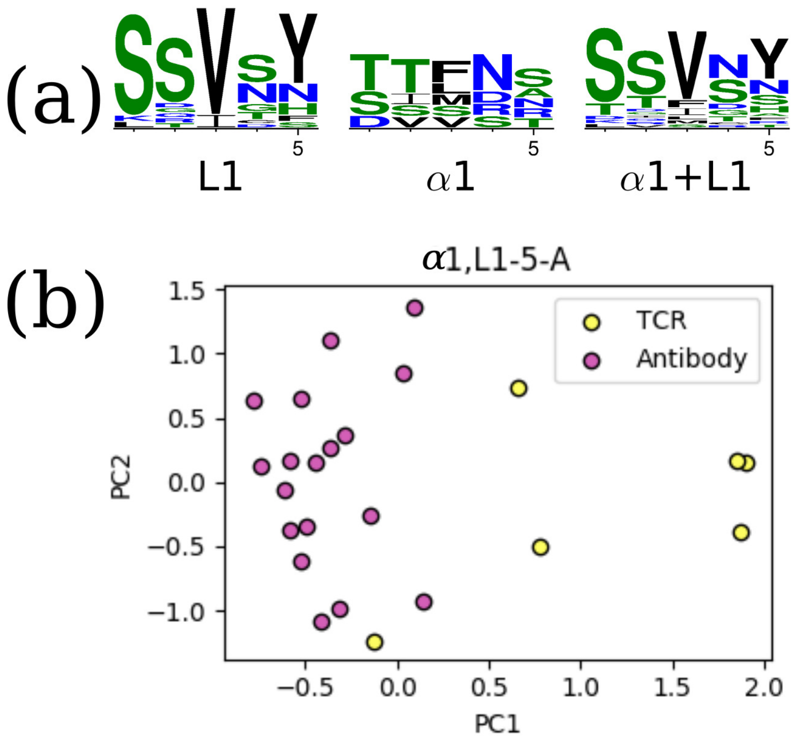

Figure S14: The unique TCR and antibody sequences in the α1,L1-5-A class. (a) Sequence logos of CDRα1 and CDRL1 loops in the α1,L1-5-A class, using only the unique sequences. The sequence patterns are distinct between the two classes. (b) Principal component analysis (PCA) plot of the first two components in one-hot-encoded sequences, stratified by TCR and antibody CDRs (see Materials and Methods). TCR α1 and antibody L1 can be separated by the first principal component. One TCR sequence (DSVNN) is close to the antibody set.

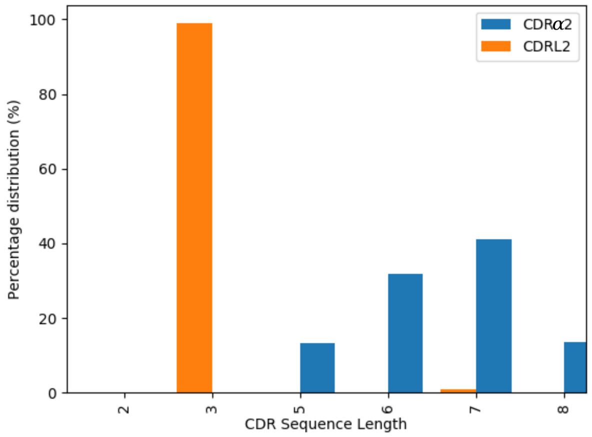

Figure S15: Length distribution of CDRα2 (blue) and CDRL2 (orange) loops. The majority of the CDRL2 loops are three-residues long, while CDRα2 adopts a range of sequence lengths.

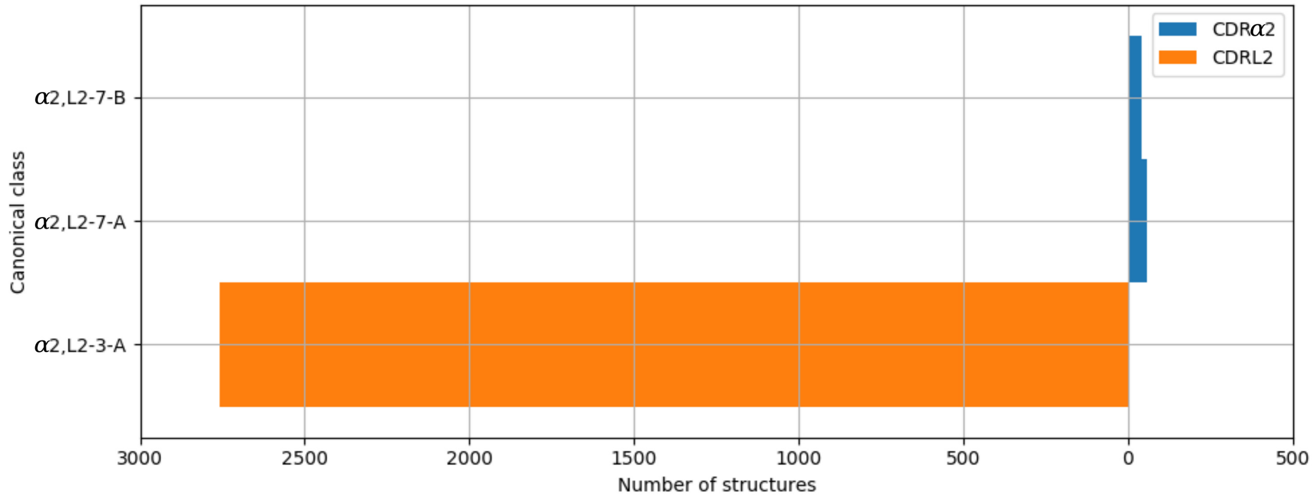

Figure S16: Clusters of CDRα2 (blue) and CDRL2 (orange) loops. Orange bars indicate the number of CDRL2 structures, and blue bars the number of CDRα2 structures. All clusters contain structures from only one CDR type, *i.e*. CDRα2 or CDRL2.

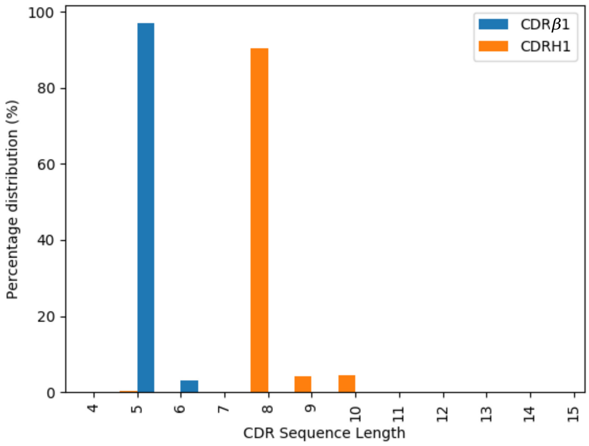

Figure S17: Length distributions of CDRβ1 (blue) and CDRH1 (orange) loops. CDRβ1 loops tend to be shorter than CDRH1 loops.

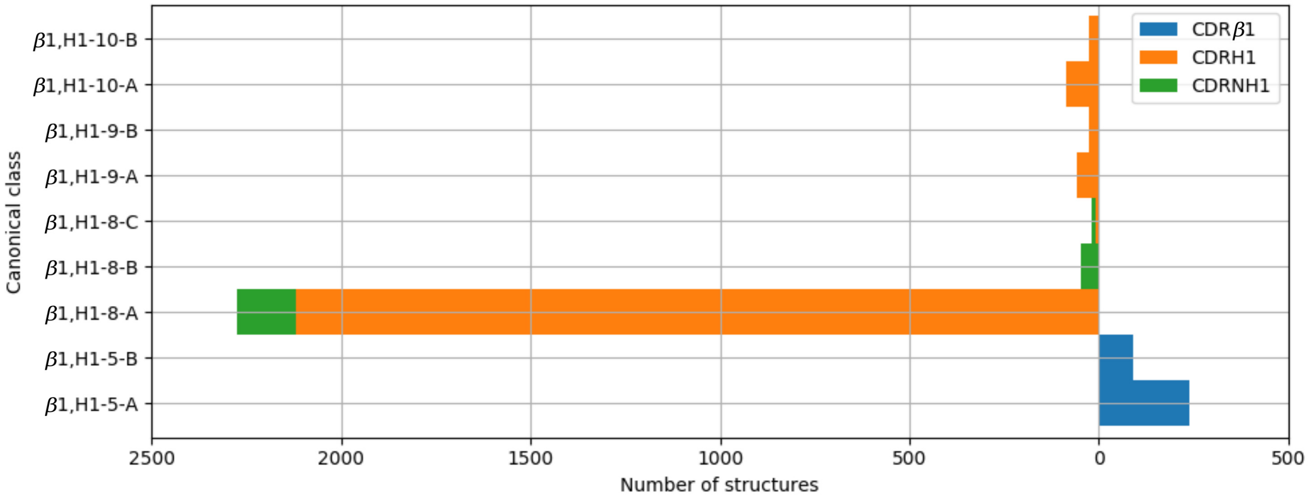

Figure S18: Clusters of CDRβ1 and CDRH1 loops. Orange bars indicate the number of antibody CDRH1 structures, green bars nanobody CDRH1 structures, and blue bars the number of CDRβ1 structures. All classes have structures from only one CDR type, *i.e.* CDRβ1 or CDRH1. Some nanobody CDRH1 loops are structurally closer to antibody CDRH1 loops than TCR CDRβ1 loops.

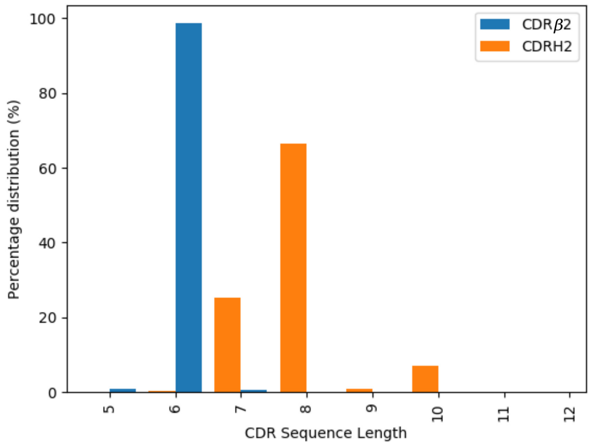

Figure S19: Length distributions of CDRβ2 (blue) and CDRH2 (orange) loops. The majority of the CDRβ2 loops are slightly shorter than CDRH2 loops.

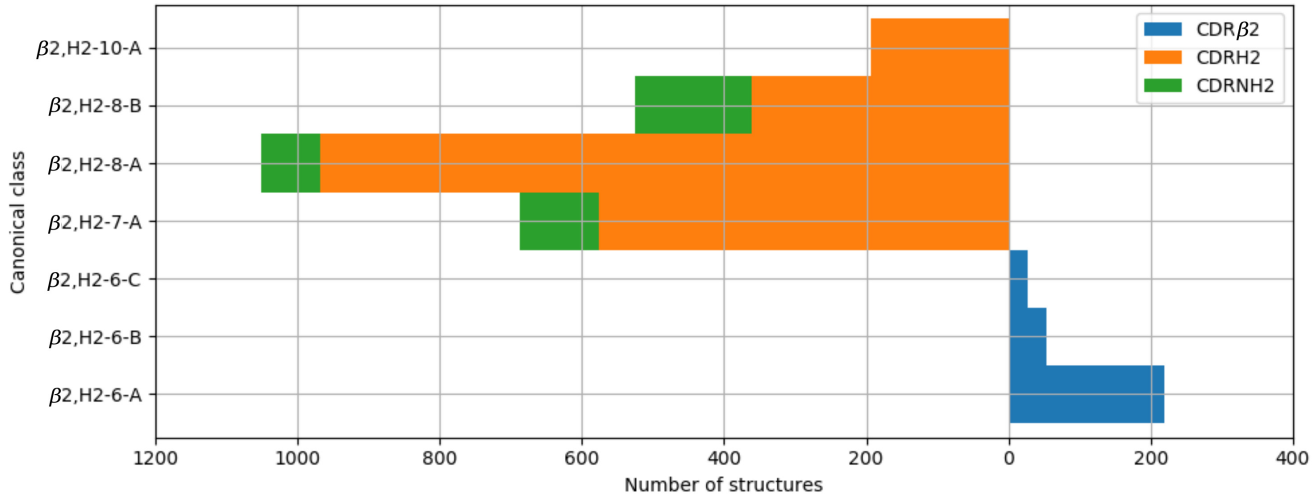

Figure S20: Clusters of CDRβ2 and CDRH2 loops. Orange bars indicate the number of antibody CDRH2 structures, green bars nanobody CDRH2 structures, and blue bars the number of CDRβ2 structures. All classes have structures from only one CDR type, *i.e.* CDRβ2 or CDRH2. Antibody and nanobody CDRH2 loops are structurally more similar than TCR CDRβ2 loops.

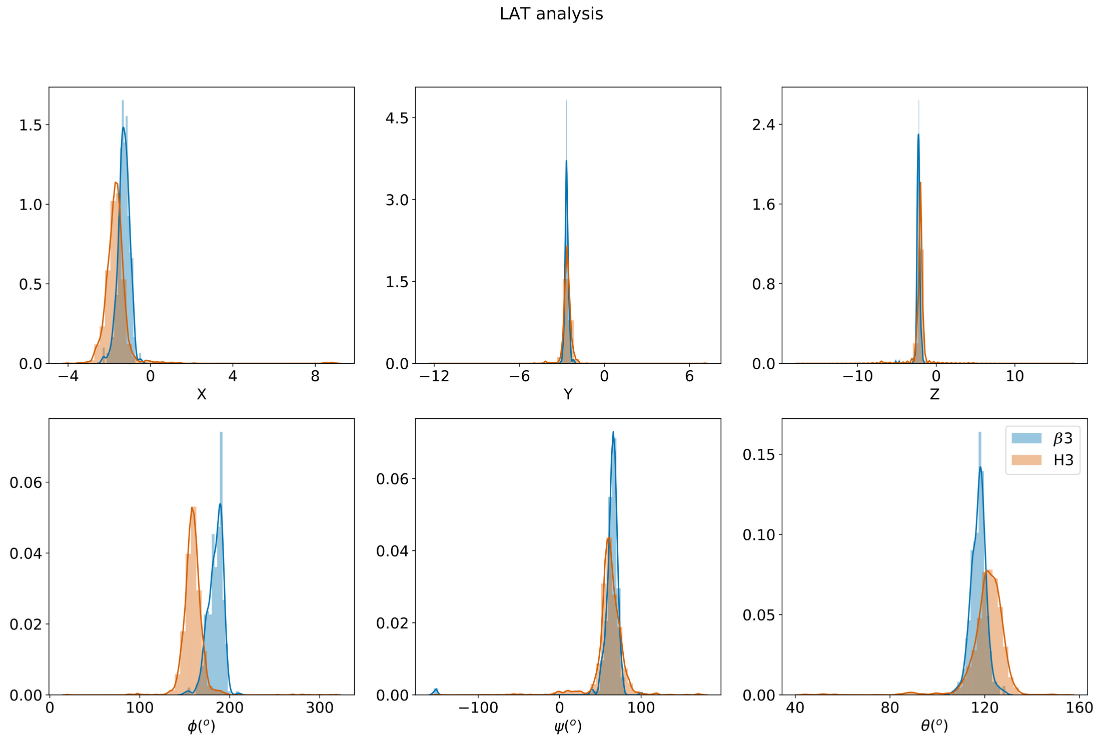

Figure S21: Loop anchor transformation (LAT) analysis of CDRβ3 and CDRH3 structures. The Euler transformation was calculated on IMGT positions 105 and 117 (see Materials and Methods). X, Y, Z, ϕ, ψ and θ are the six degrees of freedom in the Euler transformation.

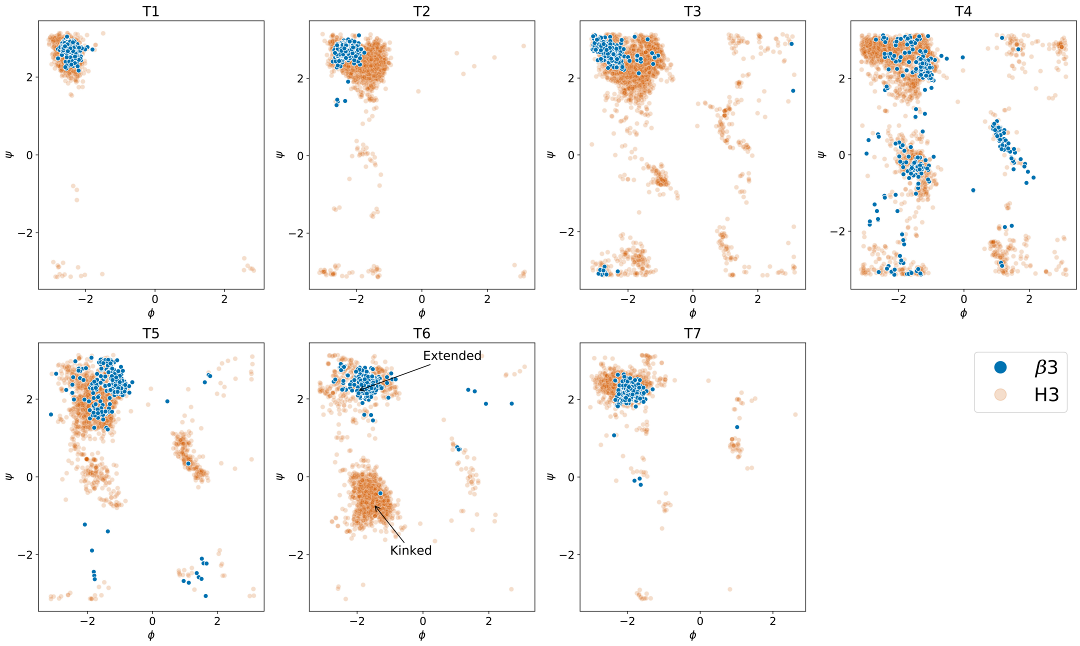

Figure S22: The ϕ/ψ plot of the first three (T1-T3, corresponding to IMGT positions 105-107) and last four (T4-T7, corresponding to IMGT positions 114-117) loop residues, in CDRβ3 and CDRH3. Residue T6 (IMGT position 116) populate different regions of the plot depending on whether the torso is extended or kinked (as labelled). See Materials and Methods and Finn et al. (2016) for details.

Table S4. TCR CDRs with multiple conformations that fall into different structural clusters (including canonical forms and pseudo-classes). 'B' and 'U' refer to the number of bound and unbound structures of the sequence within that cluster.

CDR	Sequence	Cluster 1	B	U	Cluster 2	B	U	Cluster 3	B	U
α1	ATGYPS	UC	4	-	α1,L1-6-H*	-	1	-	-	-
	DRGSQS	UC	-	2	α1,L1-6-C	16	5	-	-	-
	DRNFQY	UC	-	1	α1,L1-6-C	-	3	-	-	-
	DSAIYN	UC	1	-	α1,L1-6-J*	13	10	-	-	-
	DSSSTY	UC	1	-	α1,L1-6-C	1	-	-	-	-
	DSVNN	UC	1	-	α1,L1-5-A	3	4	α1,L1-5-B*	5	2
	YSATPY	UC	-	1	α1,L1-6-D*	4	-	α1,L1-6-E*	4	-
	VSGLRG	UC	3	-	α1,L1-6-G*	6	-	-	-	-
	TSGFNG	UC	6	4	α1,L1-6-G*	51	-	-	-	-
	TISGTDY	UC	-	1	α1,L1-7-E*	12	-	-	-	-
	SSVPPY	α1,L1-6-D*	2	5	α1,L1-6-E*	-	13	-	-	-
α2	QEAYKQQN	UC	1	-	α2,L2-8-A*	3	-	-	-	-
	ATKADDK	UC	4	-	α2,L2-7-B	2	1	-	-	-
	SSTDNKR	UC	-	2	α2,L2-7-B	-	2	-	-	-
	YYSGDPVV	UC	1	-	α2,L2-8-B*	7	1	-	-	-
	GLTSN	UC	3	1	α2,L2-5-A*	6	-	-	-	-
	GLKNN	UC	-	2	α2,L2-5-A*	4	2	-	-	-
A3	AMRGDSSYKLI	UC	-	1	α3,L3-11-B*	15	1	-	-	-
	AGAGSQGNLI	UC	1	1	α3,L3-10-H*	7	1	-	-	-
	AVNFGGGKLI	UC	-	2	α3,L3-10-J*	5	-	-	-	-
	GTYNQGGKLI	UC	2	-	α3,L3-10-A	1	-	-	-	-
	AYGEDDKII	UC	3	-	α3,L3-9-D*	2	-	-	-	-
	AVTTDSWGKLQ	UC	-	2	α3,L3-11-J*	9	-	-	-	-
	AVSESPFGNEKLT	UC	-	1	α3,L3-13-H*	2	3	-	-	-
	AVRPTSGGSYIPT	UC	-	1	α3,L3-13-C*	5	-	-	-	-
	AVRPLLDGTYIPT	UC	-	1	α3,L3-13-C*	3	-	-	-	-
β1	SEHNR	β1,H1-5-B	1	5	β1,H1-5-D*	2	1	-	-	-
	MGHDK	UC	1	-	β1,H1-5-A	7	6	-	-	-
	ENHRY	UC	2	-	β1,H1-5-A	4	1	-	-	-
β2	FQNEAQ	UC	1	-	β2,H2-6-C	8	9	-	-	-
	FNNNVP	UC	1	-	β2,H2-6-B	15	2	-	-	-
	FQNQEV	UC	-	1	β2,H2-6-D*	4	3	-	-	-
	FYNGKV	UC	-	1	β2,H2-6-B	-	3	-	-	-
	SASEGT	UC	2	-	β2,H2-6-A	47	-	-	-	-
	SFDVKD	β2,H2-6-A	-	6	β2,H2-6-F*	-	6	-	-	-
	SVGEGT	UC	-	2	β2,H2-6-A	3	3	-	-	-
	SYGAGN	UC	2	-	β2,H2-6-A	2	-	-	-	-
	SYGAGS	UC	1	-	β2,H2-6-A	34	10	-	-	-
	SYGVNS	UC	3	4	β2,H2-6-A	5	2	-	-	-
	FQGTGA	UC	1	-	β2,H2-6-C	2	-	-	-	-
	YYNGEE	UC	1	-	β2,H2-6-B	14	-	-	-	-

Table S5: Antibody CDRs with multiple conformations that fall into different structural clusters (including canonical forms and pseudo-classes). 'B' and 'U' refer to the number of bound and unbound structures of the sequence within that cluster.

CDR	Sequence	Cluster 1	B	U	Cluster 2	B	U	Cluster 3	B	U
L1	QDISNY	UC	-	1	α1,L1-6-A	30	29	-	-	-
	QDINSY	UC	2	-	α1,L1-6-A	5	2	-	-	-
	KSVSTSGYSY	UC	1	-	α1,L1-10-A	1	-	-	-	-
	KSVSTSGYNY	UC	1	-	α1,L1-10-A	1	-	-	-	-
	ESVDYYGKSF	UC	-	2	α1,L1-10-A	2	-	-	-	-
	ENVVTY	UC	-	1	α1,L1-6-A	1	3	-	-	-
	QSVSSSY	α1,L1-7-A	6	2	α1,L1-7-C*	-	3	-	-	-
	QNLLDSSFDTNT	UC	-	1	α1,L1-11,12-A	-	1	-	-	-
	QSVSSY	UC	-	3	α1,L1-6-A	11	12	-	-	-
	QSVTNY	UC	1	1	α1,L1-6-I*	2	-	-	-	-
	QSVTSSQ	UC	-	1	α1,L1-7-A	-	1	-	-	-
	SRVGY	UC	-	1	α1,L1-5-A	-	3	-	-	-
	SSDVGGYNY	UC	22	4	α1,L1-9-A	15	13	-	-	-
	SSNIGENS	UC	-	1	α1,L1-8-A	-	5	-	-	-
	QGIRND	UC	1	-	α1,L1-6-A	2	-	-	-	-
	SSVSSSY	UC	2	-	α1,L1-7-A	4	4	-	-	-
	SSVSY	UC	2	-	α1,L1-5-A	23	16	-	-	-
	SSVTY	UC	-	1	α1,L1-5-A	-	7	-	-	-
	TGAVTSGHY	UC	1	-	α1,L1-9-B	7	-	-	-	-
	SSNVGGYNY	UC	-	1	α1,L1-9-A	-	1	-	-	-
	QSIVHSNGNTY	UC	-	1	α1,L1-11,12-A	30	17	-	-	-
	QSLSSKY	UC	-	1	α1,L1-7-A	-	1	-	-	-
	QSLVHSNGNTY	UC	1	1	α1,L1-11,12-A	26	16	-	-	-
	QSVDYDGDSY	UC	-	1	α1,L1-10-A	1	3	-	-	-
	QSVSSA	UC	11	-	α1,L1-6-A	22	-	α1,L1-6-I*	3	-
L2	LTS	UC	-	1	α2,L2-3-A	5	-	-	-	-
	GNN	UC	4	-	α2,L2-3-A	12	2	-	-	-
	EVN	UC	-	1	α2,L2-3-A	35	25	-	-	-
	GAS	UC	1	-	α2,L2-3-A	101	42	-	-	-
	FTS	α2,L2-3-A	5	1	α2,L2-3-D*	-	1	-	-	-
	GKN	α2,L2-3-E*	6	1	α2,L2-3-B*	2	5	-	-	-
	DVN	UC	2	-	α2,L2-3-A	2	3	-	-	-
	YTS	UC	-	1	α2,L2-3-A	64	37	α2,L2-3-D*	1	3
	DST	UC	1	-	α2,L2-3-A	-	1	-	-	-
	SDS	α2,L2-3-A	1	-	α2,L2-3-B*	1	-	-	-	-
	DAS	UC	-	1	α2,L2-3-A	86	78	-	-	-
	GDN	α2,L2-3-A	1	-	A2,L2-3-B*	1	-	-	-	-
L3	ALWYSNHLV	UC	1	1	α3,L3-9-B	13	8	-	-	-
	YSTDSSGNHRV	UC	1	-	α3,L3-11-A	-	2	-	-	-
	SSYGGDNNLF	UC	-	1	α3,L3-10-A	-	2	-	-	-
	SSYEGSDNFV	UC	17	4	α3,L3-10-C	16	5	-	-	-
	SSRDKSGSRLSV	UC	-	1	α3,L3-12-C*	5	-	-	-	-

Continued on next page

Table S5 – continued from previous page

CDR	Sequence	Cluster 1	B	U	Cluster 2	B	U	Cluster 3	B	U
	QVYGASSYT	UC	4	-	α3,L3-9-D*	-	6	-	-	-
	QQWSSHIFT	UC	-	1	α3,L3-9-B	-	1	α3,L3-9-C	-	1
	QQWNYPFT	UC	-	1	α3,L3-8-D*	2	1	-	-	-
	QAWDASTGV	UC	1	-	α3,L3-9-G*	7	-	-	-	-
	QQHYTTPPT	α3,L3-9-A	14	18	α3,L3-9-F*	-	6	-	-	-
	QAWDSSTVV	UC	-	1	α3,L3-9-B	1	2	-	-	-
	HQYLSSWT	UC	-	1	α3,L3-8-A	-	2	-	-	-
	GVGDTIKEQFVYV	UC	2	3	α3,L3-13-F*	5	-	-	-	-
	GTWDSSLNPV	UC	-	1	α3,L3-10-A	1	1	-	-	-
	AAWDDSLDVAV	UC	-	2	α3,L3-10,11-A	-	4	-	-	-
	QQSSSSLIT	α3,L3-9-C	12	-	α3,L3-9-A	1	-	-	-	-
	FQGSHVPRT	UC	1	1	α3,L3-9-A	2	6	-	-	-
H1	RRSSRSWA	UC	-	5	β1,H1-8-D*	-	2	β1,H1-8-T*	6	-
	GRTFSSYG	UC	1	-	β1,H1-8-C	3	-	-	-	-
	GRTFSSYA	UC	-	1	β1,H1-8-A	1	-	β1,H1-8-C	1	-
	GHTYSTYC	UC	1	2	β1,H1-8-F*	2	-	-	-	-
	GGTFSSYA	UC	3	4	β1,H1-8-A	2	2	-	-	-
	GGSISTYY	UC	1	-	β1,H1-8-A	1	-	-	-	-
	GGSISSSSYY	UC	-	1	β1,H1-10-B	-	1	-	-	-
	GGSFSTYA	UC	-	3	β1,H1-8-A	28	2	-	-	-
	GRTFSSYV	β1,H1-8-A	-	1	β1,H1-8-C	7	-	-	-	-
	GGSFSSYY	UC	1	-	β1,H1-8-A	2	-	-	-	-
	GFTFSNYG	UC	1	-	β1,H1-8-A	15	4	-	-	-
	GFTFSDYD	UC	3	-	β1,H1-8-A	1	-	-	-	-
	GFSLSTSGIG	UC	1	-	β1,H1-10-A	1	-	-	-	-
	GFSLSDKA	UC	-	2	β1,H1-8-A	-	1	-	-	-
	GFSLRTSRVG	UC	1	2	β1,H1-10-C*	1	-	-	-	-
	GFSFNTNA	UC	1	1	β1,H1-8-A	2	2	-	-	-
	GFNIKDTY	UC	1	1	β1,H1-8-A	37	36	-	-	-
	GFTLDDYA	UC	1	-	β1,H1-8-A	2	-	-	-	-
	GSAVSDYA	UC	-	1	β1,H1-8-S*	-	5	-	-	-
	GSISGIVV	UC	-	1	β1,H1-8-B	3	-	-	-	-
	GSSFTGYN	UC	1	-	β1,H1-8-A	2	-	-	-	-
	GYTFTSYW	UC	4	-	β1,H1-8-A	34	30	-	-	-
	GYTFTSNW	β1,H1-8-A	1	6	β1,H1-8-C	-	1	-	-	-
	GYTFTSHW	UC	-	1	β1,H1-8-A	-	1	-	-	-
	GYTFTNYY	UC	1	1	β1,H1-8-A	4	1	-	-	-
	GYTFTNYG	UC	1	1	β1,H1-8-A	14	3	-	-	-
	GYTFTEYF	UC	-	1	β1,H1-8-A	3	-	-	-	-
	GYTFSEYW	UC	-	1	β1,H1-8-A	2	-	-	-	-
	GYSLSTSGMG	UC	-	1	β1,H1-10-A	-	1	-	-	-
	GYSITTNYA	β1,H1-9-D*	6	-	β1,H1-9-B	1	1	-	-	-
	GYSITSNYA	β1,H1-9-B	2	-	β1,H1-9-A	-	1	-	-	-
	GYSITSGYS	β1,H1-9-A	4	-	β1,H1-9-C*	3	2	-	-	-
	GYSITSDYA	UC	2	-	β1,H1-9-A	15	5	-	-	-
	GYSITSDFA	β1,H1-9-B	4	7	β1,H1-9-A	1	3	-	-	-

Continued on next page

Table S5 – continued from previous page

CDR	Sequence	Cluster 1	B	U	Cluster 2	B	U	Cluster 3	B	U
	GYSITGGYS	UC	-	2	β1,H1-9-A	-	6	-	-	-
	GYAFSSSW	UC	-	2	β1,H1-8-A	3	1	-	-	-
	GVTFSNVA	UC	1	-	β1,H1-8-A	1	-	-	-	-
	GVRLSAYD	UC	-	1	β1,H1-8-A	-	1	-	-	-
	GFNFSSSS	UC	1	-	β1,H1-8-A	2	-	-	-	-
	GDSITSGY	UC	1	-	β1,H1-8-A	17	2	-	-	-
	GSISSITT	UC	1	-	β1,H1-8-B	-	2	-	-	-
	GDSITSAY	UC	1	-	B1,H1-8-A	1	-	-	-	-
H2	ILPGSDST	UC	1	2	β2,H2-8-A	1	-	-	-	-
	IGPSGGIT	UC	-	4	β2,H2-8-B	-	4	-	-	-
	IDPSNGDT	UC	-	1	β2,H2-8-A	-	1	-	-	-
	IDPNSGGT	UC	2	-	β2,H2-8-A	11	8	-	-	-
	IDPNGGGT	UC	1	-	β2,H2-8-A	6	1	-	-	-
	IDPEQGNT	UC	-	2	β2,H2-8-A	1	1	-	-	-
	ISAGGDKT	UC	2	-	β2,H2-8-B	2	2	-	-	-
	IGSSGGQT	β2,H2-8-B	1	-	β2,H2-8-C*	-	1	-	-	-
	ISASGGST	β2,H2-8-B	-	4	β2,H2-8-G*	2	-	-	-	-
	ISGSGGNT	UC	1	-	β2,H2-8-B	-	1	-	-	-
	FNPSNGRT	UC	-	1	β2,H2-8-A	-	1	-	-	-
	ISGSGGST	UC	1	-	β2,H2-8-A	-	2	β2,H2-8-B	25	21
	ASNGGII	UC	-	1	β2,H2-7-A	-	1	-	-	-
	ISNLDGST	UC	2	1	β2,H2-8-A	-	1	-	-	-
	ISPGNGDI	UC	-	1	β2,H2-8-A	2	-	-	-	-
	ISPYSGVT	UC	1	-	β2,H2-8-A	1	1	-	-	-
	ISGGGRNI	β2,H2-8-B	-	1	β2,H2-8-L*	4	-	-	-	-
	IGTSGNI	UC	1	1	β2,H2-7-A	4	1	-	-	-
	IHYRGTT	UC	-	1	β2,H2-7-A	1	1	-	-	-
	IIPIFGTA	UC	3	2	β2,H2-8-A	4	6	-	-	-
	IRSKSINSAT	UC	1	-	β2,H2-10-A	-	3	-	-	-
	IRSGGGRT	UC	3	-	β2,H2-8-B	1	-	-	-	-
	IRNKPYNYET	UC	1	-	β2,H2-10-A	2	-	-	-	-
	INTHSGVP	UC	1	-	β2,H2-8-A	1	-	-	-	-
	INSNGGST	UC	1	-	β2,H2-8-B	6	1	-	-	-
	INPGSDYT	UC	2	-	β2,H2-8-A	2	1	-	-	-
	INPGNGYT	UC	-	2	β2,H2-8-A	2	1	-	-	-
	INLNGGRT	UC	-	1	β2,H2-8-A	-	1	-	-	-
	INIYTGEP	UC	1	-	β2,H2-8-A	2	1	-	-	-
	INIGATYA	UC	1	-	β2,H2-8-B	1	-	-	-	-
	ILPGSGST	UC	3	-	β2,H2-8-A	4	5	-	-	-
	ILPGSGRT	UC	2	-	β2,H2-8-A	1	1	-	-	-
	ILPGGGSN	UC	-	2	β2,H2-8-A	-	2	-	-	-
	IKSKTDGGTT	UC	-	1	β2,H2-10-A	-	5	-	-	-
	IIPILGIA	UC	1	-	β2,H2-8-A	1	-	-	-	-
	ISRSGSVT	UC	1	-	β2,H2-8-B	2	1	-	-	-
	IRSSVIT	UC	1	-	β2,H2-7-B*	6	1	-	-	-
	ISSGGGNT	UC	-	2	β2,H2-8-B	1	-	-	-	-

Continued on next page

Table S5 – continued from previous page

CDR	Sequence	Cluster 1	B	U	Cluster 2	B	U	Cluster 3	B	U
	ISSPGTI	UC	-	3	β2,H2-7-A	-	1	-	-	-
	VIPLLTIT	UC	4	-	β2,H2-8-A	25	6	-	-	-
	TNPRNGGT	UC	1	-	β2,H2-8-A	4	2	-	-	-
	TIPLFGKT	UC	-	1	β2,H2-8-A	1	1	-	-	-
	NNPGNGYI	UC	-	1	β2,H2-8-A	-	1	-	-	-
	MSHEGDKT	UC	-	1	β2,H2-8-B	-	1	-	-	-
	MDSGGGGT	UC	9	1	β2,H2-8-B	-	1	-	-	-
	LTTTGTA	β2,H2-7-A	3	1	β2,H2-7-C*	1	4	-	-	-
	IYWDDVE	UC	-	1	β2,H2-7-A	1	-	-	-	-
	IYWDDDK	UC	1	-	β2,H2-7-A	8	7	-	-	-
	IYSYYGST	UC	2	-	β2,H2-8-A	2	-	-	-	-
	IYSSYSYT	UC	1	-	β2,H2-8-A	1	-	-	-	-
	ISSGGGRT	UC	1	1	β2,H2-8-B	1	-	-	-	-
	IYPYYGST	β2,H2-8-A	1	-	β2,H2-8-D*	3	-	-	-	-
	IYPYSGST	β2,H2-8-A	2	-	β2,H2-8-D*	1	-	-	-	-
	IYRSGSRM	UC	-	1	β2,H2-8-B	-	1	-	-	-
	IYPTNGYT	UC	1	-	β2,H2-8-A	16	28	-	-	-
	ISWSGGST	UC	-	1	β2,H2-8-B	1	1	-	-	-
	ITGPGEGWSV	UC	1	-	β2,H2-10-A	9	-	-	-	-
	ITPAGGYT	UC	3	-	β2,H2-8-J*	-	18	-	-	-
	VYDSGDT	UC	-	3	β2,H2-7-A	-	1	-	-	-
	IWAGGST	UC	2	-	β2,H2-7-A	-	1	-	-	-
	IWGDGIT	UC	-	1	β2,H2-7-A	1	-	-	-	-
	ITYSGTT	UC	1	1	β2,H2-7-A	1	-	-	-	-
	IWSGGST	UC	-	1	β2,H2-7-A	7	10	-	-	-
	IWWDDDN	UC	1	-	β2,H2-7-A	1	-	-	-	-
	IWYSGSNT	UC	-	2	β2,H2-8-C*	-	4	β2,H2-8-K*	-	9
	IYPGNGDT	UC	1	-	β2,H2-8-A	4	11	-	-	-
	IYPGNVHA	UC	-	1	β2,H2-8-A	-	2	-	-	-
	IYPGSGGT	UC	-	1	β2,H2-8-A	-	2	-	-	-
	IWPSGGNT	UC	1	-	β2,H2-8-B	1	1	-	-	-
	IRWNGGST	UC	1	-	β2,H2-8-B	2	-	-	-	-

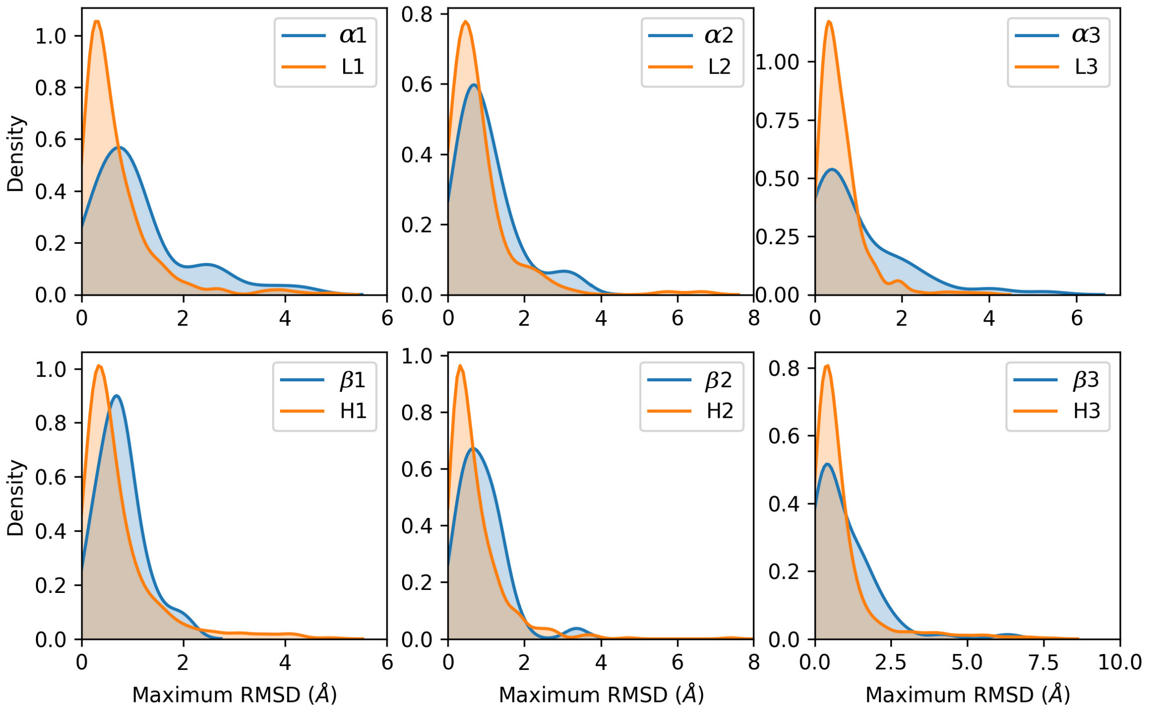

Figure S23: The density of the maximum root-mean square deviation (RMSD) between loop structures with identical CDR sequence. TCR CDRs (blue) show a shift to the right compared to antibody CDRs (orange), suggesting that structural flexibility of TCR CDRs is greater than that of antibodies.

REFERENCES

Berman HM, Westbrook J, Feng Z, Gilliland G, Bhat TN, Weissig H, et al. The Protein Data Bank. *Nucleic Acids Res* **28** (2000) 235–242.

Crooks GE, Hon G, Chandonia JM, Brenner SE. Weblogo: a sequence logo generator. *Genome research* **14** (2004) 1188–1190.

Al-Lazikani B, Lesk AM, Chothia C. Canonical structures for the hypervariable regions of t cell $\alpha\beta$ receptors. *J Mol Biol* **295** (2000) 979–995.

Klausen MS, Anderson MV, Jespersen MC, Nielsen M, Marcatili P. LYRA, a webserver for lymphocyte receptor structural modeling. *Nucleic Acids Res* **43** (2015) W349–W355.

Maceiras A, Almeida S, Mariotti-Ferrandiz E, Chaara W, Six A, Hori S, et al. T follicular helper and t follicular regulatory cells have different tcr specificity. *Nat Commun* **8** (2017).

Finn JA, Leman JK, Willis JR, Cisneros III A, Crowe Jr JE, Meiler J. Improving loop modeling of the antibody complementarity-determining region 3 using knowledge-based restraints. *PloS one* **11** (2016) e0154811.
